# Inhibition of insulin-like growth factor-1 receptor signaling enhances growth-inhibitory and proapoptotic effects of gefitinib (Iressa) in human breast cancer cells

**DOI:** 10.1186/bcr1028

**Published:** 2005-04-12

**Authors:** Anne Camirand, Mahvash Zakikhani, Fiona Young, Michael Pollak

**Affiliations:** 1 Lady Davis Institute for Medical Research and Department of Oncology, McGill University, Montréal, QC, Canada; 2 Biology Department, Faculty of Science, Az-Zahra University, Vanak, Tehran, Iran

## Abstract

**Introduction:**

Gefitinib (Iressa, ZD 1839, AstraZeneca) blocks the tyrosine kinase activity of the epidermal growth factor receptor (EGFR) and inhibits proliferation of several human cancer cell types including breast cancer. Phase II clinical trials with gefitinib monotherapy showed an objective response of 9 to 19% in non-small-cell lung cancer patients and less than 10% for breast cancer, and phase III results have indicated no benefit of gefitinib in combination with chemotherapy over chemotherapy alone. In order to improve the antineoplastic activity of gefitinib, we investigated the effects of blocking the signalling of the insulin-like growth factor 1 receptor (IGF-1R), a tyrosine kinase with a crucial role in malignancy that is coexpressed with EGFR in most human primary breast carcinomas.

**Methods:**

AG1024 (an inhibitor of IGF-1R) was used with gefitinib for treatment of MDA468, MDA231, SK-BR-3, and MCF-7 breast cancer lines, which express similar levels of IGF-1R but varying levels of EGFR. Proliferation assays, apoptosis induction studies, and Western blot analyses were conducted with cells treated with AG1024 and gefitinib as single agents and in combination.

**Results:**

Gefitinib and AG1024 reduced proliferation in all lines when used as single agents, and when used in combination revealed an additive-to-synergistic effect on cell growth inhibition. Flow cytometry measurements of cells stained with annexin V-propidium iodide and cells stained for caspase-3 activation indicated that adding an IGF-1R-targeting strategy to gefitinib results in higher levels of apoptosis than are achieved with gefitinib alone. Gefitinib either reduced or completely inhibited p42/p44 Erk kinase phosphorylation, depending on the cell line, while Akt phosphorylation was reduced by a combination of the two agents. Overexpression of IGF-1R in SK-BR-3 cells was sufficient to cause a marked enhancement in gefitinib resistance.

**Conclusion:**

These results indicate that IGF-1R signaling reduces the antiproliferative effects of gefitinib in several breast cancer cell lines, and that the addition of an anti-IGF-1R strategy to gefitinib treatment may be more effective than a single-agent approach.

## Introduction

The signaling activity of receptor protein tyrosine kinases (PTKs) is crucial to the control of apoptosis, differentiation, and proliferation processes; consequently, dysfunction or deregulation of these molecules can lead to uncontrolled growth and neoplastic progression. The abnormal activation of PTKs in the pathology of many cancers has called attention to these receptors as potential targets for therapeutic intervention [1-4]. Some neoplastic conditions arise from excessive activity of a single PTK, for example Bcr-Abl in chronic myeloid leukaemia [5], or c-kit or platelet-derived growth factor receptor-α in gastrointestinal stromal cell tumours [6], and these conditions are effectively treated using the PTK inhibitor Gleevec (Imatinib mesylate) [7]. However, most cancers have complex biochemical causes and may involve dysfunction of several PTKs as well as crosstalk between downstream signaling pathways. One approach to address the multiplicity problem involves cotargeting different PTKs [8-17], but for maximal efficacy, the choice of PTKs to be simultaneously blocked in any specific cancer type is crucial.

The epidermal growth factor receptor (EGFR, erbB1, or HER1) is a 170-kDa member of the erbB family of PTKs, which are transmembrane receptors with important roles in development, differentiation, proliferation, and migration [18]. The activation of EGFR by ligand binding causes dimerization and autophosphorylation of the receptor and subsequent recruitment of downstream molecules, leading to mitogenic signaling [19]. EGFR is overexpressed in a large subset of primary breast carcinomas, and EGFR ligands such as TGF-α and amphiregulin are found in 50 to 90% of primary breast carcinomas [20]. The co-occurrence of these sets of factors is associated with poor prognosis and resistance to hormonal therapy [21].

Several anti-EGFR molecules have been shown to cause neoplastic growth inhibition [22]. Among these, gefitinib (Iressa; AstraZeneca) is an orally active synthetic anilinoquinazoline (4-(3-chloro-4-fluroanilino)-7-methoxy-6-(3-morpholinopropoxy) quinazoline) that inhibits EGFR but also has activity against erbB2 and vascular endothelial growth factor receptor 2 (VEGFR-2) at 100-fold higher than those needed for EGFR inhibition [23]. It has proved an effective inhibitor of proliferation in experimental human breast cancer cell systems, either alone or in combination with other antineoplastic agents [10,11,14,24-32]. Gefitinib as second- or third-line monotherapy in phase II trials of non-small-cell lung cancer patients provided objective tumour response rates of 9 to 19% [22,33,34]. A response rate of 10.8% was also seen in head and neck cancer patients [35], but phase II trials in advanced breast cancer patients showed partial response in fewer than 10% of patients [36-38]. Non-small-cell lung cancer phase III trials where gefitinib was used in combination with traditional chemotherapy (paclitaxel, gemcitabine, or cisplatin) showed no added benefit of gefitinib to patients over chemotherapy alone [39,40]. The acceptable safety profile of gefitinib was, however, confirmed by these studies, and the results motivate studies to determine if PTK cotargeting might improve the efficacy of the drug.

A potential cotarget receptor in breast cancer is the insulin-like growth factor 1 receptor (IGF-1R). In its mature form, IGF-1R is a heterotetrameric receptor (two extracellular 125-kDa α chains and two transmembrane 95-kDa β chains) that autophosphorylates after ligand binding and activates several downstream signaling routes, including the phosphatidylinositol 3-kinase (PI3K) and mitogen-activated protein kinase (MAPK) pathways. Signaling through IGF-1R stimulates proliferation, promotes angiogenesis and metastasis, and inhibits apoptosis [41-45]. There is now abundant evidence indicating that signaling through the IGF-1R pathway is important in many cancers, including breast cancer [4,42,46-49], and recent preclinical work has shown that IGF-1R could be used as a successful cotarget with EGFR in primary human glioblastoma cells [13,50], with c-kit in small-cell lung cancer [15,17], and with HER2/erbB2 in breast cancer cells [12,16]. AG1024 (3-bromo-5-*t*-butyl-4-hydroxy-benzylidenemalonitrile) is a synthetic tyrphostin that inhibits ligand-stimulated IGF-1R autophosphorylation in intact cells, with an inhibitory concentration 50% (IC_50_) of 7 μM and can affect the insulin receptor at 9- to 10-fold higher concentrations (IC_50 _57 μM) [51]. Tyrphostins bind to the active site of receptors and modify its conformation to prevent the substrate and ATP from binding [51]. Through its anti-IGF-1R activity, AG1024 inhibits cell proliferation and induces apoptosis in several cell systems, including non-small-cell lung cancer [52], small-cell lung cancer [17], melanoma [53], and breast cancer [54].

In this study, AG1024 and gefitinib were used to cotarget IGF-1R and EGFR activity in several human breast cancer cell lines that express IGF-1R similarly but present different levels of EGFR. We show that combination treatment causes additivity or synergy in growth inhibition and apoptosis induction, and we speculate that adding an anti-IGF-1R strategy to gefitinib treatment may be more effective than single-agent gefitinib therapy.

## Materials and methods

### Chemicals and drugs

Gefitinib (ZD 1839, Iressa) was a gift from AstraZeneca (Macclesfield, UK). AG1024 was purchased from Calbiochem-EMD Biosciences (La Jolla, CA, USA).

### Cell lines and proliferation assays

Breast cancer cell lines MCF-7, MDA468, MDA231, and SK-BR-3 were obtained from ATCC (Manassas, VA, USA). Cells were cultured at 37°C with 5% CO_2 _in RPMI 1640 (MCF-7) or McCoy medium (all other cell lines) with 10% fetal bovine serum (FBS) (InVitrogen, Gaithersburg, MD, USA), except in growth inhibition assays, where the FBS supplement was reduced to 1%. Cell proliferation was measured with the Alamar Blue dye reduction method (Biosource International, Camarillo, CA, USA). Growth tests were conducted with 10^4 ^cells/well in 200 μl media in 96-well plates, and three replicates per dose combination were used for each experiment. Experiments shown here are representative of three repeats. Stock solutions of tyrphostin AG1024 and gefitinib were made in dimethyl sulfoxide to 10 mM, stored at -20°C, and diluted in medium containing 1% FBS just before use. The concentration of dimethyl sulfoxide in the final culture was kept below 0.2% (v/v). All procedures involving tyrphostins were conducted in low light intensity.

### Flow cytometry for receptors

Medium was removed from breast cancer cells growing in monolayers, and cells were collected by scraping in 1 ml 4°C FACS (fluorescence-activated cell sorter) buffer (3% fetal bovine serum, 0.02% NaN_3 _in PBS). Cells were centrifuged and washed in FACS buffer; approximately 10^6 ^cells were stained with phycoerythrin-conjugated anti-IGF-1R (BD Pharmingen, San Diego, CA, USA), or with fluorescein-isothiocyanate-conjugated (FITC-conjugated) anti-EGFR antibody (Santa Cruz Biotechnology, Santa Cruz, CA, USA) for 30 min at 4°C in the dark, washed twice in FACS, and resuspended in the same buffer. Analysis was conducted for 20,000 cells using a FACSCalibur flow cytometer (BD Biosciences, Burlington, MA, USA) with CellQuest software (BD Biosciences Immunocytometry Systems, Franklin Lakes, NJ, USA). Normal mouse IgG_1 _(Santa Cruz Biotechnology) was used for isotype determination. All tests were conducted in duplicate and the experiments shown here are representative of two repeats.

### Flow cytometry for apoptosis induction

Growth medium was removed from breast cancer cells growing in monolayers; adherent cells were briefly trypsinized, detached, combined with floating cells from the original growth medium, centrifuged, and washed twice with PBS. Approximately 10^6 ^cells were stained for 30 min with annexinV–FITC and propidium iodide using the ApoTarget kit (Biosource International). Analysis was conducted on a FACSCalibur flow cytometer using CellQuest software (see receptor section, above). For quantification of caspase-3 activation, cells (approximately 0.5 × 10^6^) were obtained as for testing with annexinV and propidium iodide, but were washed in media, resuspended in 150 μl media containing 10% FBS and 0.5 μl Red-DEVD-FMK (Caspase-3 detection kit, Calbiochem-EMD Biosciences), and incubated for 30 min at 37°C in a cell-culture incubator with 5% CO_2_. The stained cells were centrifuged, washed twice with the wash buffer provided in the kit, resuspended in 500 μl of the same buffer, and analyzed for fluorescence on a FACSCalibur flow cytometer using CellQuest software. All apoptosis tests were conducted in duplicate and results shown are representative of three experiments.

### Western blotting and immunoprecipitation

Cells growing in monolayers in 10-cm culture plates were treated with various doses of AG1024, gefitinib, or vehicle for 24 or 72 hours, then lysed in nondenaturing buffer (1% Nonidet NP-40, 20 mM TrisCl pH 8.0; 0.5 mM sodium orthovanadate, pH 9.0; and proteinase inhibitors (Roche, Mannheim, Germany)), and particulate material was removed by centrifugation at 4°C. Samples (50 μg) of the supernatant were separated on 10% or 15% polyacrylamide gels. After transfer to TransBlot nitrocellulose membranes (BioRad, Hercules, CA, USA), the proteins were reacted overnight with the following primary antibodies at 1:1,000 dilution: anti-Akt, anti-phospho-Akt (Ser473), anti-Erk1/Erk2 (p44/42) anti-phospho-Erk1/Erk2 (Thr202/Tyr204), and anti-EGFR (Cell Signalling Technologies, Beverly, MA, USA). Anti-phospho-EGFR (Tyr1173) was from Upstate (Charlottesville, VA, USA). Blots were then reacted for 1 hour with 1:2,000 horseradish-peroxidase-conjugated antirabbit immunoglobulin G (Pharmacia-Amersham, Piscataway, NJ, USA). Tubulin 1:200 (Santa Cruz Biotechnology) and antimouse immunoglobulin G (Pharmacia-Amersham) were used to check evenness of loading. Membranes were reacted with enhanced chemiluminescence (ECL) reagents (Pharmacia-Amersham) and exposed to X-OMAT LS film (Kodak, Rochester, NJ, USA). For immunoprecipitation, 500-μg samples of soluble protein in a final volume of 500 μl were incubated with 10 μl antiphosphotyrosine monoclonal antibody (BD Pharmingen, Mississauga, ON, Canada) with rotation at 4°C overnight. A mixture (20 μl) of protein A and G+ Sepharose beads (Santa Cruz Biotechnology) was then added, and the samples were rotated at 4°C for 1 hour. Beads were collected by centrifugation, washed once with lysis buffer, heated for 5 min at 95°C in SDS–PAGE loading buffer, and separated by electrophoresis. Membranes after transfer were reacted with an anti-IGF-1R β-subunit antibody (Santa Cruz Biotechnology) and processed as above for enhanced chemiluminescence detection. Western blot analyses were repeated twice.

### Statistical analysis

Statistical validity was evaluated using Student's *t*-test or the Student Newmany–Keuls test for multiple pairwise comparisons of means with Statistical Analysis System software, version 8 (SAS Institute, Cary, NC, USA), with *P *values ≤ 0.05 considered significant.

## Results

### Surface expression of IGF-1R and EGFR in breast cancer cell lines

**Figure 1:**
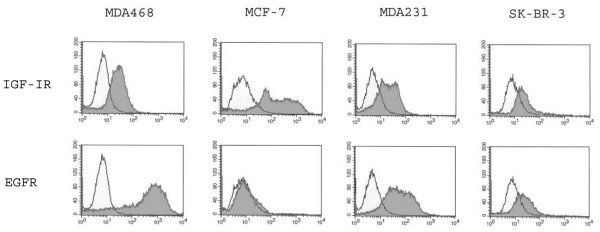
Surface expression of IGF-1R and EGFR in human breast cancer cell lines. Untreated cells were stained with phycoerythrin-conjugated anti-IGF-1R (insulin like growth factor-1 receptor) or with fluorescein-isothiocyanate-conjugated anti-EGFR (anti-epidermal growth factor receptor) antibody. Shaded peaks show flow cytometry analysis of the number of insulin-like growth factor 1 (top row) and epidermal growth factor (bottom row) receptors on the surface of MDA468, MCF-7, MDA231, and SK-BR-3 human breast cancer cells. Outlined peak represents isotype control (normal mouse IgG_1_). Counts indicate number of events.

The breast cancer cell lines tested exhibit similar surface expression of the IGF-1 receptor, but the number of EGF receptors varied considerably, with MDA468 cells showing very high expression, MDA231 intermediate levels, SK-BR-3 low expression, and MCF-7 no significant presence of EGFR (Fig. 1).

### Inhibition of IGF-1R signaling enhances the effect of gefitinib on the proliferation of breast cancer cell lines

In the culture conditions used here, proliferation IC_50 _values (means ± standard deviations) for AG1024 were 3.5 μM ± 0.4 for MDA468; 3.5 μM ± 0.5 for MCF-7; 4.5 μM ± 0.4 for MDA231; and 2.5 ± 0.4 for SK-BR-3 cells. The respective IC_50 _values for gefitinib were 8.0 μM ± 1.0; 9.2 μM ± 2.3; 11.5 μM ± 3.0; and 6.5 μM ± 1.5.

**Figure 2:**
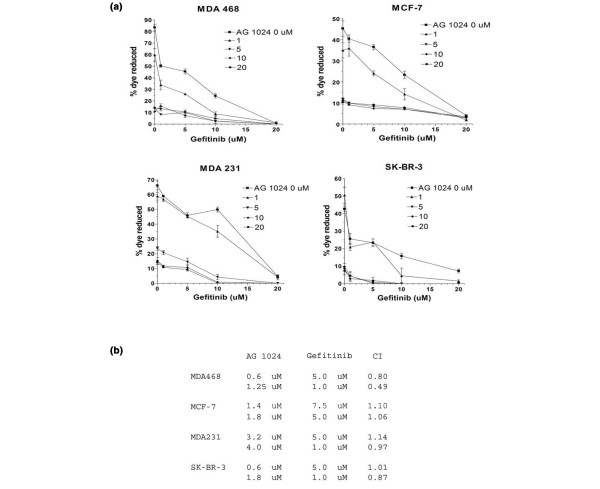
Inhibition of breast cancer cell growth by AG1024 and gefitinib singly and in combination. **(a) **Cells in exponential stages of growth were exposed to increasing concentrations of inhibitors for 72 hours in media containing 1% fetal bovine serum. Triplicates were used for each dose combination for each experiment. **(b) **Proliferation combination index (CI) values were calculated using the classic isobologram equation [55] and indicate synergy (CI < 1) or additivity (CI approximately 1).

The use of treatments combining AG1024 and gefitinib revealed that the cotargeting approach achieved a greater growth inhibition (Fig. 2a). Combination index (CI) values calculated according to the classic isobologram equation [55] evaluate the interactions between agents as additive (CI approximately 1), antagonistic (CI >1), or synergistic (CI <1). The results (Fig. 2b) indicate synergy (for MDA468) or additivity (other cell lines) of interaction between AG1024 and gefitinib.

### Adding an anti-IGF-1R strategy to gefitinib treatment increases levels of apoptosis

**Figure 3:**
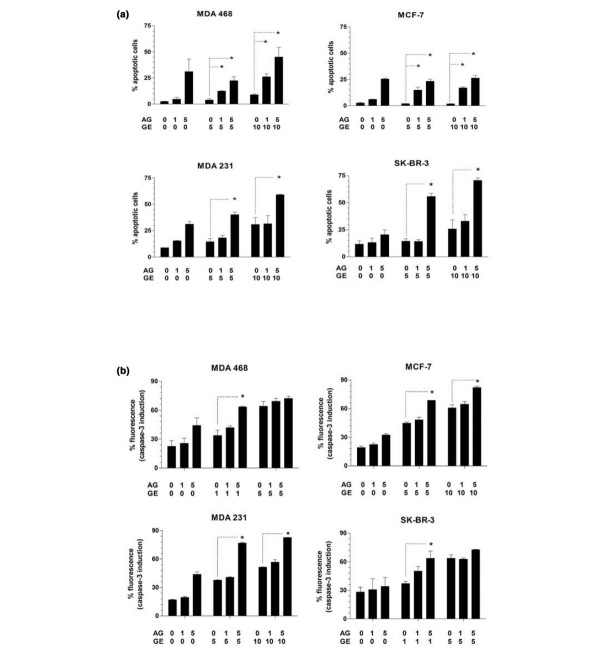
Treatment with AG1024 enhances apoptotic effects of gefitinib. **(a) **Flow cytometric analysis of apoptosis in cells stained with annexin V and propidium iodide after 72-hour treatment of breast cancer cells with AG1024 (AG), gefitinib (GE), or both. **(b) **Flow cytometric analysis of caspase-3 induction by Red-DEVD-FMK fluorescence after 24-hour treatment of cells with AG1024, gefitinib, or both. Addition of AG1024 to gefitinib treatment significantly enhanced apoptotic induction over levels achieved by gefitinib alone. Values on horizontal axes are concentrations (μM). *P < 0.05.

Flow cytometric analyses of breast cancer cells treated with AG1024, gefitinib, or both, and stained with annexinV and propidium iodide (cells treated for 3 days) or with red–DEVD–FMK for caspase-3 activation (cells treated for 1 day) are shown in Fig. 3a,b. In all cell lines, and for both methods of detecting apoptosis, conditions were found where addition of AG1024 significantly increased apoptosis levels over those seen with gefitinib alone.

### Effect of treatment with AG1024 or gefitinib on protein and phosphorylation levels of Akt and p44/p42 Erk kinases

**Figure 4:**
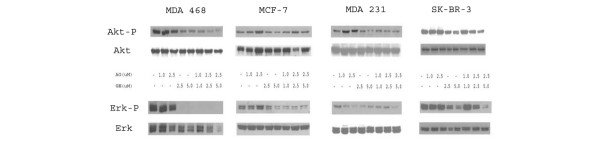
Effect of treatment on Erk and Akt kinases phosphorylation and protein levels. Western blot analysis showing phosphorylation (P) (top) and protein (bottom) levels of Akt and p44/p42 Erk kinases in cells treated for 24 hours with AG1024 (AG), gefitinib (GE), or both.

After 24 hours of treatment, gefitinib decreased the levels of Erk phosphorylation in most cell lines, and completely eliminated Erk phosphorylation in MDA468 (Fig. 4). In contrast, the phosphorylation levels of Akt were reduced by the combination of the two agents. Erk and Akt protein levels were not affected by the 24-hour treatments. Tubulin levels confirmed equal loading (not shown).

### Overexpression of IGF-1R greatly reduces sensitivity to gefitinib

**Figure 5:**
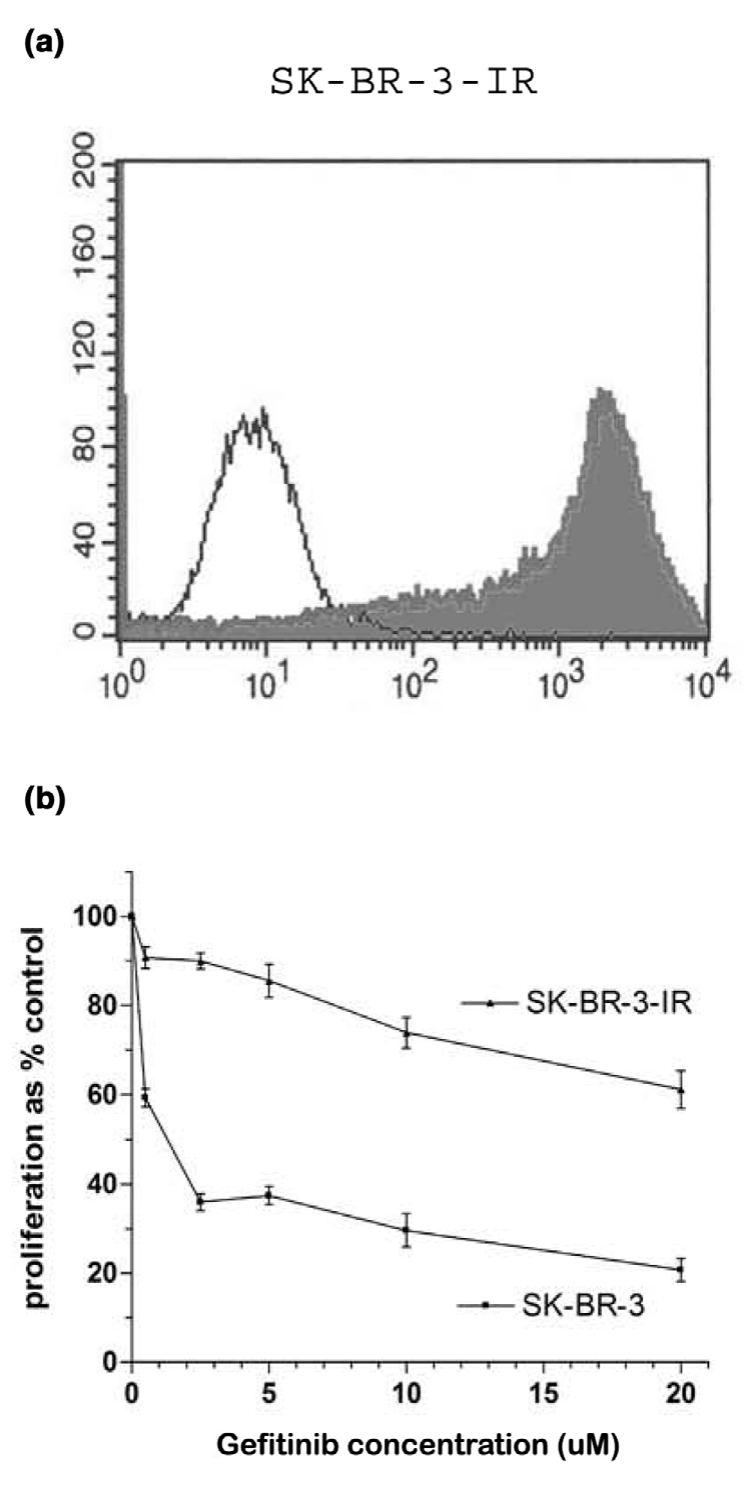
Overexpression of IGF-1R results in enhanced resistance to gefitinib antiproliferation activity. **(a) **Flow cytometry estimation of surface expression levels of epidermal growth factor 1 receptor (IGF-1R) (shaded peak) in the SK-BR-3-IR line, which differs from its parental line, SK-BR-3, only by the transfected IGF-1R gene [12]. Outlined peak is normal mouse IgG_1 _isotype. Counts indicate number of events. **(b) **Effect of a 72-hour treatment with gefitinib alone on the proliferation of SK-BR-3 and SK-BR-3-IR cells. Triplicates were used for each dose.

SK-BR-3 cells transfected to overexpress the IGF-1 receptor (SK-BR-3-IR) [12] were tested for sensitivity to gefitinib. Figure 5a illustrates the high IGF-1R expression levels observed by flow cytometry in SK-BR-3-IR cells compared with the levels in the SK-BR-3 parental line shown in Fig. 1. Increased expression of IGF-1R caused a very marked enhancement in resistance to the growth-inhibitory effects of gefitinib (Fig. 5b).

### Effect of treatment with AG1024 or gefitinib on tyrosine phosphorylation of IGF-1R and EGFR

**Figure 6:**
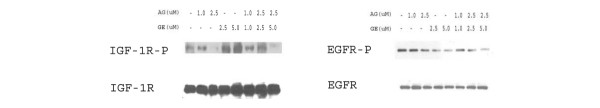
Single agent treatment effect on tyrosine phosphorylation (P; top) and protein expression of receptors. Western blot showing phosphorylation (P, top) and receptor protein levels (bottom). MCF-7 cells were used for detection of phosphorylation of the insulin like growth factor 1 receptor (IGF-1R) and MDA468 cells, for phosphorylation analysis of the epidermal growth factor receptor (EGFR). AG, AG1024; GE, gefitinib.

An example of the effect of AG1024, gefitinib, or both (24-hour treatment) on the phosphorylation levels of IGF-1 and EGF receptors in 1% serum conditions is illustrated in Fig. 6. In MCF-7 cells (left), AG1024 at 2.5 μM eliminated phosphorylation of IGF-1R, while gefitinib did not affect the phosphorylation state of IGF-1R. EGFR phosphorylation levels were decreased by gefitinib, but only slightly affected by AG1024 treatment (MDA468 cells, Fig. 6, right). Protein levels for both receptors were unaffected by treatment in the conditions used here.

## Discussion

Several reports have suggested that cotargeting protein tyrosine kinases results in substantial enhancement of growth inhibition [8-17]. In the present study, the choice of the IGF-1 receptor as cotarget is based on the knowledge that this receptor drives important cell survival pathways [41-45] and that reduction of its antiapoptotic effects increases the efficacy of treatments targeting several other neoplasia-related PTKs [12,13,15-17]. The results presented here show the effects of adding an anti-IGF-1R strategy to gefitinib treatment in human breast cancer cell lines chosen for their similar expression of IGF-1R but their different EGFR levels (Fig. 1).

Gefitinib and AG1024 used as single agents show antiproliferative activity on all cell lines tested, and their combination produces an additive-to-synergistic enhancement of growth inhibition (Fig. 2a,b). The mechanism of action on cancer cells of EGFR blockers such as AG 1478, mAb225, and gefitinib is generally cytostatic and proceeds via a G0/G1 arrest [56]. Most breast cancer cells are growth-arrested by gefitinib, but only a subset shows induction of apoptosis (cytotoxic effect) [31], and high doses of the drug are needed to induce apoptosis in normal mammary epithelial cells and primary cultures of mammary carcinoma cells [24]. Blocking the antiapoptotic IGF-1R pathway with AG1024 improves apoptosis induction over the level due to treatment with gefitinib alone (Fig. 3). All the cell lines tested exhibited this effect, regardless of the levels of expression of EGFR. In fact, the growth-inhibitory effect of gefitinib has been reported to be independent of the levels of expression of EGFR in human breast cancer cells [10,24-26,31] and other cancer cell lines [57]. As the EGFR expression level is not a good predictor of gefitinib sensitivity [58], EGFR expression status in tumours cannot be used to exclude patients from gefitinib trials [59]. It has been shown that the presence of somatic mutations in the EGFR gene in lung cancer samples correlates with sensitivity to gefitinib [60,61]. However, even in the absence of detectable EGFR (as in MCF-7 cells: our results, Fig. 1, and [10]), gefitinib and AG1024 still have additive capability, raising the possiblity of a non-EGFR-specific gefitinib effect that can be enhanced by the anti-IGF-1R agent.

Western blot analysis (Fig. 4) showed that after a 24-hour treatment, gefitinib affects phosphorylation levels of p44/p42 Erk and Akt kinases, but that combination treatment with the anti-IGF-1R agent causes a further reduction in levels of Akt phosphorylation. The effect is particularly visible for MDA468 cells, which probably reflects the fact that these cells show a synergistic rather than additive growth reduction pattern. Interestingly, MDA468 cells (PTEN-null) (phosphatase-and-tensin-homolog-null) have been reported to show a relative resistance to gefitinib that can be reversed through the use of the PI3K inhibitor LY294002 [62] or PTEN reconstitution [30], pointing to a crucial role for receptors that signal through the PI3K cascade, such as IGF-1R. MDA468 cells are also the most sensitive to gefitinib inhibition of Erk phosphorylation. In longer treatments (not shown), the levels of protein expression for Akt and Erk are decreased by AG1024 or by the combination of agents. AG1024 treatment has been reported to decrease the expression of several proteins known as regulators of apoptosis and the cell cycle [53,54], and the inhibitor may therefore also provide a longer-term inhibitory effect by mechanisms involving protein degradation.

An important point, illustrated in Fig. 5, is that overexpression of the IGF-1 receptor results in increased resistance to gefitinib. This observation implies that one way in which breast cancer cells resist gefitinib is through the signaling activity of IGF-1R. Since gefitinib does not affect phosphorylation of the IGF-1 receptor (Fig. 6 and [63]), our results suggest that the antiapoptotic pathways driven by IGF-1 signalling should be targeted in order to optimize the antineoplastic effects of gefitinib. While our model system involves increased IGF-1R activity due to receptor overexpression, it must be noted that increased IGF-1R signaling in clinical breast cancer might also arise from mechanisms involving abnormally high IGF-2 expression or from derangements in IGF-binding protein physiology [42].

The findings described here suggest that the antineoplastic effects of gefitinib may be significantly underestimated if examined only under conditions in which IGF-IR is fully functional. Several anti-IGF-1R compounds are now being developed for clinical evaluation [64-67], and it should soon be feasible to conduct trials to test the hypothesis that the efficacy of gefitinib treatments is enhanced by IGF-1R targeting. The data presented here support further research into breast cancer therapeutic strategies combining gefitinib with anti-IGF-1R agents.

## Conclusion

In several human breast cancer cell lines, addition of the IGF-1R inhibitor AG1024 to gefitinib reduced cell proliferation in an additive or synergistic fashion and enhanced the induction of apoptosis over levels achieved by gefitinib alone. This effect was independent of levels of expression of the EGF receptor. Overexpression of IGF-1R in SK-BR-3 cells was sufficient to cause a marked enhancement in gefitinib resistance. IGF-1R signaling can therefore limit the antiproliferative effects of gefitinib *in vitro*, and we speculate that for a subset of human breast cancers, adding an anti-IGF-1R strategy to gefitinib treatment may be more effective than a single-agent approach.

## Abbreviations

CI = combination index; EGFR = epidermal growth factor receptor; FACS = fluorescence-activated cell sorter; FBS = fetal bovine serum; FITC = fluorescein isothiocyanate; IC_50 _= inhibitory concentration 50%; IGF-1R = insulin like growth factor 1 receptor; PI3K = phosphatidylinositol 3-kinase; PTEN = phosphatase and tensin homolog; PTK = protein tyrosine kinase; VEGFR = vascular endothelial growth factor receptor.

## Competing interests

The author(s) declare that they have no competing interests.

## Authors' contributions

Anne Camirand: study design, data collection, statistical analysis, data interpretation, manuscript preparation, literature search and funds collection. Mahvash Zakikhani: data collection, statistical analysis, data interpretation, manuscript preparation, literature search. Fiona Young: data collection. Michael Pollak: study design, funds collection.

All authors read and approved the final manuscript.
